# Divergence in cell cycle progression is associated with shifted phenology in a multivoltine moth: the European corn borer, *Ostrinia nubilalis*

**DOI:** 10.1242/jeb.245244

**Published:** 2023-06-09

**Authors:** Qinwen Xia, Chao Chen, Erik B. Dopman, Daniel A. Hahn

**Affiliations:** ^1^ Entomology and Nematology Department, University of Florida, Gainesville, FL 32611, USA; ^2^ Department of Biology, Tufts University, Medford, MA 02155, USA

**Keywords:** Cell cycle, Diapause, Phenology, Gene expression

## Abstract

Evolutionary change in diapause timing can be an adaptive response to changing seasonality, and even result in ecological speciation. However, the molecular and cellular mechanisms regulating shifts in diapause timing remain poorly understood. One of the hallmarks of diapause is a massive slowdown in the cell cycle of target organs such as the brain and primordial imaginal structures, and resumption of cell cycle proliferation is an indication of diapause termination and resumption of development. Characterizing cell cycle parameters between lineages differing in diapause life history timing may help identify molecular mechanisms associated with alterations of diapause timing. We tested the extent to which progression of the cell cycle differs across diapause between two genetically distinct European corn borer strains that differ in their seasonal diapause timing. We show the cell cycle slows down during larval diapause with a significant decrease in the proportion of cells in S phase. Brain–subesophageal complex cells slow primarily in G0/G1 phase whereas most wing disc cells are in G2 phase. Diapausing larvae of the earlier emerging, bivoltine E-strain (BE) suppressed cell cycle progression less than the later emerging, univoltine Z-strain (UZ) individuals, with a greater proportion of cells in S phase across both tissues during diapause. Additionally, resumption of cell cycle proliferation occurred earlier in the BE strain than in the UZ strain after exposure to diapause-terminating conditions. We propose that regulation of cell cycle progression rates ultimately drives differences in larval diapause termination, and adult emergence timing, between early- and late-emerging European corn borer strains.

## Teaser

**Summary:** Regulation of cell cycle progression rates drives differences in larval diapause termination, and adult emergence timing, between early- and late-emerging European corn borer strains.

## INTRODUCTION

Diapause is a developmental strategy that synchronizes life cycles with predictable seasonal changes in the biotic and abiotic environments (Tauber and Tauber, 1981; Danks, 1987). Many insect species enter diapause as a response to a combination of token stimuli (e.g. photoperiod and temperature) prior to the onset of adverse conditions. Specifically, many insects in temperate regions must successfully complete each of the following developmental phases to precisely coordinate diapause: (1) begin the diapause preparatory phase in response to token stimuli that induce diapause, often photoperiodic cues; (2) initiate diapause by triggering a switch from continuous growth and reproduction to a massive slowdown of the life cycle to allow the insects to enter their programmed dormancy before the onset of seasonally stressful conditions; (3) maintain diapause to avoid lethal temperatures or lack of resources; and (4) terminate diapause and subsequently resume development to coordinate their life cycle with the return of favorable seasonal conditions (Koštál, 2006).

However, long standing seasonal patterns can be altered by anthropogenic climate change, urbanization, species introductions, host-plant shifts and an array of other biotic and abiotic factors (Bradshaw and Holzapfel, 2001; Filchak et al., 2000; IPCC, 2014; Williams et al., 2015). In response to altered seasonality, diapause can evolve rapidly by shifting the timing of the onset of diapause initiation, diapause termination, or both (Filchak et al., 2000; Bradshaw and Holzapfel, 2001, 2006; Gomi et al., 2007; van Asch et al., 2013). Subtle shifts in the timing of diapause can substantially alter annual life-history patterns and have big impacts on the synchronization between insect life cycles and environmental conditions (Wadsworth and Dopman, 2015; Denlinger et al., 2017). For example, under the warmer spring conditions resulting from anthropogenic climate change, hatching of winter moth *Operophtera brumata* eggs showed decreased synchrony with the bud burst of its host oak *Quercus robur* (Visser and Holleman, 2001). After a decade, however, van Asch et al. (2013) showed that the egg hatch of winter moths became more synchronized with oak bud burst by delaying the timing of termination of their egg diapause. Hence, rapid evolution of diapause timing can be an adaptive response to environmental change, and similar changes in diapause timing have been shown in a number of systems (Bradshaw and Holzapfel, 2001; Gomi et al., 2007; van Asch et al., 2013).

To better understand and predict how organisms may respond to novel seasonal environments, numerous studies have focused on exploring the mechanisms underlying shifts in diapause timing (Bradshaw and Holzapfel, 2001; Ragland et al., 2011; van Asch et al., 2013; Wadsworth and Dopman, 2015; Kozak et al., 2019). Although identification of specific genes controlling diapause timing has been challenging (Feder et al., 2003; Wadsworth and Dopman, 2015; Wadsworth et al., 2015), some candidate genes involved in diapause regulation and seasonal timing have been identified. For example, in the tephritid fruit fly *Rhagoletis pomonella*, genes in the Wnt and TOR signaling pathways are very promising candidates for regulating the termination of pupal diapause (Dowle et al., 2020; Ragland et al., 2011). In the cotton bollworm *Helicoverpa armigera*, cell cycle regulation and stress resistance genes have been proposed to be important for diapause initiation (Bao and Xu, 2011). As one of the cues for diapause regulation, seasonal changes in day length are monitored by a seasonal clock that is associated with components of the circadian clock (Bradshaw and Holzapfel, 2009; Denlinger et al., 2017). Allelic variation in several circadian clock genes has been associated with seasonal adaptation and diapause incidence (Pegoraro et al., 2014; Schmidt et al., 2008; Tauber et al., 2007; Yamada and Yamamoto, 2011). For example, in the European corn borer, *Ostrinia nubilalis*, polymorphism in the clock gene *period* lies under a major quantitative trait locus (QTL) peak determining variation in diapause termination time and post-diapause development time, as does allelic variation in a second circadian-related gene, the *pigment-dispersing factor receptor*, that lies under a separate but interacting QTL peak (Kozak et al., 2019; Levy et al., 2015, 2018; Wadsworth and Dopman, 2015). However, it is still largely unknown how the regulation of diapause-related genes is changed by alterations in seasonal information and subsequently transduced into ecologically relevant variation in diapause timing (Ragland et al., 2019).

One of the hallmarks of diapause is a massive slowdown in the cell cycle and the resumption of cell cycle proliferation is a clear indication of diapause termination (Denlinger, 2002; Koštál et al., 2009; Podrabsky and Culpepper, 2012; Ragland et al., 2011; Shimizu et al., 2016; Shingleton et al., 2003; Tammariello and Denlinger, 1998). Therefore, characterizing cell cycle parameters between lineages that differ in diapause life-history timing may help us to identify molecular mechanisms underlying alterations of diapause timing as a response to changing seasonality. The cell cycle consists of four distinct phases: G0/G1, S, G2 and M (Norbury and Nurse, 1992; Vermeulen et al., 2003). G0 phase is a resting stage where the cell is not actively growing or dividing. G0 is distinguished from G1 phase, during which cells are actively growing, and synthesizing RNA, proteins and other biomolecules in preparation for the next phase, DNA synthesis (S phase). During S phase, genomic DNA is copied in preparation for cell division (G2 phase). During G2 phase, cells evaluate whether there are errors in the duplicated chromosomes and make the necessary repairs before the genetic material is passed on to a daughter cell. During the subsequent mitotic (M) phase, cells divide into two daughter cells (Canaud and Bonventre, 2015). At G1 and G2, cells can either continue into the next phase of the cell cycle or undergo cell cycle arrest. The transitions between cell cycle phases are tightly regulated by two key sets of proteins that regulate cell cycling: cyclins and cyclin-dependent kinases (Cdks), which are highly conserved across organisms (Schafer, 1998). Specifically, cyclin D binds to Cdk4/6 to regulate progression through G1 phase. Cyclin E and cyclin A bind to Cdk2 to initiate the transition into S phase and to regulate progression through S phase, respectively. Then, cyclin B binds to Cdk1 to drive entry from G2 into M phase (Poon, 2002). The mechanistic regulation of progression from one cell cycle stage to the next is highly conserved among animals, from *Drosophila melanogaster* and *Caenorhabditis elegans* to mice and humans (Gönczy, 2008; Noatynska et al., 2013).

For individuals undergoing diapause, cell division and differentiation are either significantly slowed or completely arrested in target organs such as primordial imaginal structures and the central nervous system (CNS), preventing further development (Tammariello and Denlinger, 1998). Therefore, cell cycle slowdown or arrest in target tissues is one of the hallmarks of diapause. Cell cycle slowdown has been widely studied in plant dormancy (Velappan et al., 2017), *Caenorhabditis elegans* dauer larvae (van den Heuvel, 2005) and annual killifish embryos (Podrabsky and Culpepper, 2012). However, to our knowledge there are only six reports describing cell cycle parameters in diapausing insects, covering six different species with substantial differences in their diapause life histories, making a synthesis of cell cycle progression, slowdown and arrest with respect to the phases of insect diapause a challenge. In embryonic diapause of the silkworm *Bombyx mori*, 98% of cells across the whole embryo are in G2 phase of the cell cycle (Nakagaki et al., 1991). In contrast, in the band-legged ground cricket, *Dianemobius nigrofasciatus*, more than 90% of diapausing egg cells are in G0/G1 phase during embryonic diapause (Shimizu et al., 2018). Similarly, in diapausing pupae of the flesh fly *Sarcophaga crassipalpis*, 97% of brain cells are in G0/G1 phase (Tammariello and Denlinger, 1998). During the pupal diapause of the tobacco hornworm, *Manduca sexta*, cells of the optic lobe are largely in G2 phase of the cell cycle (Champlin and Truman, 1998). In the diapausing larvae of the drosophilid fly *Chymomyza costata*, 86.6% of the CNS cells are in G0/G1, and 12.8% are in G2 phase (Koštál et al., 2009). Similarly, in the larval diapause of the jewel wasp *Nasonia vitripennis*, approximately 80% and 20% of brain cells arrest their cell cycle in the G0/G1 and G2 phases, respectively (Shimizu et al., 2016). From these few studies, it can been seen that there is no clear pattern; some species report cellular slowdown predominantly in G0/G1 phase and some report it in G2 phase during diapause. Some species such as flesh flies and silkworms have almost all cells in one phase of the cell cycle, but other species such as the jewel wasp and *C. costata* have most cells in the G0/G1 stage, but with a substantial portion also in the G2 stage. What proximate mechanisms and ultimate selective forces may drive these patterns of cell cycle stages during diapause among species is currently underexplored. Furthermore, because the sampling is so sparse and studies have not investigated the same tissues or life stages across species, there is no clear consensus about whether certain tissues within a diapausing insect arrest in one cell cycle stage or another. Perhaps tissues differ in their regulatory architecture for cell cycle arrest during diapause, but before one can speculate about the proximate and ultimate mechanisms that may regulate cell cycle arrest across tissues or across species, more work across life stages and tissues within species is clearly needed.

Here, we used two genetically distinct strains of the European corn borer, *Ostrinia nubilalis*, that differ in their diapause-timing characteristics to study associations between the cell cycle and shifts in seasonal life-history timing via diapause regulation. We measured cell cycle status with flow cytometry in the brain–subesophageal ganglion (SG) complex and the wing discs because cellular proliferation within these two important tissues is synonymous with continuous development, whereas a massive slowdown is synonymous with larval diapause (Koštál et al., 2009). To explore potential molecular mechanisms underlying divergence in cell cycle slowdown between the two strains, transcript abundance of four cell cycle regulators (*cyclin A*, *cyclin B*, *cyclin D* and *cyclin E*), *Proliferating cell nuclear antigen* (*PCNA*), transcription factor 1 (*E2F1*) and *polo* were investigated. PCNA is a regulator of DNA synthesis, and its expression is controlled by E2F1 transcription factor-containing complexes (Mansilla et al., 2020).

In upstate New York, USA, where the two strains originated, the E strain of *Ostrinia nubilalis* is bivoltine with one generation that occurs at the beginning of June and another generation that occurs at the end of August, while the Z strain is univoltine and has a single generation in the middle of July (Dopman et al., 2010). Wadsworth et al. (2013) showed that a 1 month shift in the life cycle of the earlier emerging bivoltine, E-strain (BE) from that of the later emerging univoltine, Z-strain (UZ) of *O. nubilalis* is the result of advancing the timing of diapause termination in the spring. We hypothesized that cell cycle progression would be associated with divergence in life cycle timing between these two strains of *O. nubilalis*. Within our broader hypothesis, we tested three specific predictions. First, in diapausing larvae of *O. nubilalis*, S phase of the cell cycle will be more suppressed in the wing discs than in the brain–SG complex because the brain–SG complex is the main tissue regulating diapause maintenance and termination (Williams, 1946), and thus may be more active in development during diapause than the wing discs. Second, the longer diapausing UZ strain will suppress S phase of cell cycle more than the shorter diapausing BE strain during the diapause maintenance stage of *O. nubilalis*. Although diapause is often considered a state of developmental arrest, development can still progress during diapause, but just at a very low rate (Shingleton et al., 2003). Therefore, we expected that the shorter diapausing BE strain would show greater levels of cell cycling during diapause than the longer diapausing UZ strain, even though both strains would show a massive reduction in cell cycling during diapause compared with their non-diapausing counterparts. Third, the shorter-diapausing BE strain will resume substantial cell cycling earlier than the longer diapausing UZ strain after transfer of both strains to conditions that are favorable for diapause termination.

We found cell cycle slowdown in both G0/G1 and G2 phases during larval diapause, in agreement with observations of larval diapause in *C. costata* and *N. vitripennis*. However, the percentage of cells in G0/G1 and G2 phases was very different between the brain–SG complex and wing discs in both strains. During diapause, the proportion of cells in S phase was significantly lower in the wing discs than in the brain–SG complex. We also found that diapausing BE strain individuals suppressed cell cycle division less than diapausing UZ strain individuals in both brain–SG complexes and wing discs during the diapause maintenance stage, which is associated with faster and earlier development of the BE strain than the UZ strain in spring. Additionally, as expected, the resumption of cell cycle proliferation occurred earlier in the earlier emerging BE strain than in the later emerging UZ strain of *O. nubilalis* after exposure to diapause-terminating conditions.

## MATERIALS AND METHODS

### Insects and sampling

**Fig. 1.**
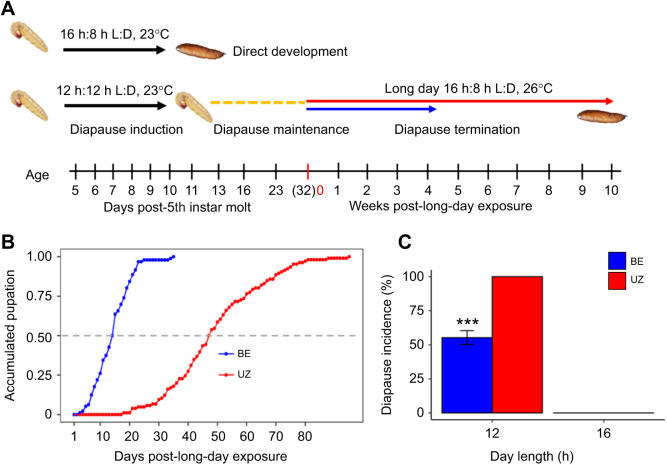
**Developmental trajectories of the earlier emerging BE strain and later emerging UZ strain of *Ostrinia nubilalis*.** (A) Schematic overview of the experimental design and time line. Diapause induction and maintenance phases were timed by the number of days after individuals molted into the 5th instar. At day 32 after the 5th instar molt, diapausing larvae were transferred to the long-day condition for diapause termination. The phase of diapause termination was timed by the number of weeks after long-day exposure. The day of transfer from diapause-maintaining, short-day conditions (12 h:12 h light:dark, L:D, 23°C) to diapause-terminating, long-day conditions (16 h:8 h L:D, 26°C) was recorded as week 0. The blue and red arrows represent the earlier emerging BE strain and the later emerging UZ strain, respectively. (B) Accumulated pupation after exposing diapausing larvae to long-day conditions: 50% of larvae of the BE and UZ strains pupated on day 12 and day 46 after transfer, respectively (*n*=95). (C) Mean±s.e.m. diapause incidence induced under short day length in BE and UZ strains of *O. nubilalis*. Short-day treatment (12 h:12 h L:D at 23±1°C) induced 55.3% and 100% diapausing larvae in the BE and UZ strains, respectively (*n*=124). ***P<0.001.

The BE and UZ strains of *Ostrinia nubilalis* (Hübner 1796) originated from colonies maintained at Tufts University. Both strains were originally collected from the field in upstate New York and kept under mass-rearing conditions in the laboratory (Glover et al., 1992). After egg hatching, larvae were fed on an artificial diet (lot no. 052418ECB, Southland Products Inc., Lake Village, AR, USA) in plastic deli-cups (11.75 cm top diameter, 9.84 cm bottom diameter and 7.62 cm height; Bare by Solo, Dart Container Corporation, Mason, MI, USA) for active growth. Non-diapausing individuals were obtained by rearing under long-day conditions including a 16 h:8 h light:dark (L:D) cycle at 23±1°C. A short-day photoperiod 12 h:12 h L:D at a constant 23±1°C was used for larval diapause induction. Some proportions of short-day larvae do not enter diapause, or they have a diapause period too brief to distinguish them from non-diapause larvae. Thus, larvae were determined to be in diapause after they remained as post-feeding 5th instar larvae when kept on a 12 h:12 h L:D cycle at 23±1°C for 32 days (Fig. 1A). In contrast, non-diapausing individuals pupated at approximately day 11 and emerged as moths at approximately day 20 after the 5th instar molt. Larvae that were clearly in diapause 32 days after the 5th instar molt were individually transferred from their rearing container into a new chamber that was modified from a 1 ml pipette tip containing cotton moistened with water to track diapause termination. By using only larvae that were clearly in diapause after 32 days, we eliminated any individuals that did not enter diapause. No food was needed in the diapause termination chamber because diapausing larvae do not feed during diapause or during larval–pupal metamorphosis. Diapausing larvae are sensitive to long-day and high-temperature cues for the termination of diapause and resumption of development from larvae into pupal morphogenesis; thus, we shifted both temperature and light cycle to strongly trigger larval diapause termination. Specifically, 32 days after 5th instar molt in diapause-induction conditions (12 h:12 h L:D cycle at 23±1°C), diapausing larvae were transferred to a 16 h:8 h L:D cycle at 26±1°C to trigger diapause termination.

The brain–SG tissue complex and both pairs of wing discs were dissected from the same individual larva in ice-cold 1× PBS buffer and stored separately in 500 µl storage buffer provided with the Cycle TEST Plus DNA Reagent Kit (Becton Dickinson, San Jose, CA, USA) at −20°C for flow cytometric analysis. We included 6 individual larval replicates for each stage and strain as described below (*n*=6 per strain and time point). Diapausing and non-diapausing tissue samples were collected on days 5, 6, 7, 8, 9 and 10 after the molt into the 5th instar. Note that non-diapausing individuals stop feeding and become prepupae at day 10 after molting into the 5th instar. To continue checking the cell cycle status of diapausing larvae before transferring them to diapause termination conditions, additional samples on days 11, 13, 16 and 23 after the 5th instar molt were also collected for diapausing larvae.

To describe cell cycle parameters during diapause termination, in the shorter diapausing BE strain, brain–SG complexes and wing discs were collected on the day of transfer into the diapause-terminating long-day conditions, as well as 1, 2 and 3 weeks (days 39, 46 and 53 after 5th instar molt) after being transferred into long-day conditions. Note that 78.6% of diapausing BE individuals had terminated diapause and initiated larval–pupal metamorphosis by 3 weeks after transfer to long-day, diapause-terminating conditions (Fig. 1B). In the UZ strain, samples were collected at the time of transfer into diapause-terminating conditions as well at 1, 2, 3, 4, 5, 6, 7, 8, 9 and 10 weeks (days 39, 46, 53, 60, 67, 74, 81, 88, 95 and 102 after 5th instar molt) after transfer to long-day conditions because of their longer diapause duration. Note that 80% of diapausing UZ individuals had terminated larval diapause and pupated by 10 weeks after transfer to long-day, diapause-terminating conditions (Fig. 1B).

To associate the photoperiodic effect on cell cycle status with transcript abundance of a series of cell cycle regulators (*cyclin A*, *cyclin B*, *cyclin D*, *cyclin E*, *E2F1*, *PCNA* and *polo*), brain–SG complexes of long-day (days 5, 6, 7, 8, 9 and 10 after the 5th instar molt) and short-day (days 5, 6, 7, 8, 9, 10, 16 and 23 after the 5th instar molt) 5th instar larvae of UZ strain of *O. nubilalis* were sampled. Brain–SG complexes of UZ and BE strain *O. nubilalis* larvae during diapause maintenance (10, 16, 23 and 32 days after the 5th instar molt) and termination phases (39, 46, 53, 60, 67, 74, 81, 88, 95 and 102 days after the 5th instar molt) were also sampled to reveal strain differences in transcript abundance of cell cycle regulators.

### Flow cytometry analysis

The preparation of uniform suspensions of single nuclei was conducted according to the protocol supplied with the Cycle TEST Plus DNA Reagent Kit (Becton Dickinson) with subtle modifications. Specifically, after thawing the sample at room temperature and spinning it down at 12,000 ***g*** at 4°C for 5 min, the storage buffer was removed and a volume of 125 µl trypsin buffer was added and mixed by gently tapping the sample tube by hand. The sample was allowed to react with the trypsin buffer for 10 min at room temperature, then 100 µl trypsin inhibitor and RNase-containing buffer was added and the sample tube was again gently tapped by hand to mix and incubated for 10 min at room temperature. Then, 100 µl propidium iodide stain solution was added and incubated for at least 10 min in the dark on ice. Finally, to avoid clogging from cell and tissue fragments, the samples were filtered through 50 µm nylon mesh before flow cytometry analysis. Cellular DNA content was analyzed using an Accuri C6 Flow Cytometer (Becton Dickinson). For each sample replicate, 10,000 nuclei were analyzed using ModFit LT4.1 software (Verity Software House, Topsham, ME, USA) and the cells were classified as being in G0/G1, S and G2/M phase depending on the intensity of the fluorescence peaks (Crissman et al., 1975).

### RNA extraction and qPCR

RNA from brain–SG complexes and wing discs was extracted using the Ambion RNAqueous-Micro Kit (cat. no. AM 1931, Thermo Fisher Scientific, Waltham, MA, USA) following the manufacturer's protocol. cDNA was synthesized from 500 ng of total RNA using a High-Capacity cDNA Reverse Transcription Kit (cat. no. 4368814, Thermo Fisher Scientific) according to the manufacturer's protocol. qPCR was conducted on a Thermal Cycler CFX96 Real-Time system (Bio-Rad, Hercules, CA, USA) using SsoAdvanced Universal SYBR Green Supermix (cat. no. 1725271, Bio-Rad). *Ribosomal protein S03* (*RpS03*) served as an internal reference standard. Primers for *PCNA*, *RpS03*, *cyclin A*, *cyclin B*, *cyclin D*, *cyclin E*, *E2F1* and *polo* were designed from the European corn borer transcriptome (Wadsworth and Dopman, 2015) and can be found in Table S1. Amplified fragments were approximately 100 bp in length. The PCR product of each transcript was sent to Genewiz (South Plainfield, NJ, USA) for Sanger sequencing to confirm amplicon identity. These sequences showed identity to European corn borer transcriptomic data in a Blast search (NCBI). Relative quantification of a target gene to a reference gene was done according to Pfaffl (2001). We conducted RNA preparation using a pool of tissues from seven individuals as a single replicate, and we repeated the procedure for a total of three replicates.

### Statistical analysis

Proportions of cells in any cell phase and transcript abundance data were analyzed using beta regression with the betareg model followed by *post hoc* Tukey's HSD tests for comparing among multiple groups in R (i386 3.5.0). First, to identify potential strain differences in cell cycle progression and transcript abundance under diapause maintenance and termination conditions, the percentage of cells in each cell cycle phase as well as transcript abundance before and after the switch to long-day conditions was analyzed between the BE and UZ strains. The day of sampling (days 10, 16, 23 and 32 for diapause maintenance phase, days 39, 46 and 53 for termination phase) and strain (UZ and BE) were treated as two fixed factors. Second, the effect of photoperiod on cell cycle progression and transcript abundance was analyzed by comparing the percentage of cells in each cell cycle phase and transcript abundance between diapause-destined and continuously developing non-diapause 5th instar larvae. Photoperiod (long-day versus short day) and larval age (developmental time) were treated as fixed factors. Only the time points where data were collected for both photoperiodic regimes (12 h:12 h and 16 h:8 h L:D) were included in these analyses (days 5, 6, 7, 8, 9 and 10 after the 5th instar molt). Third, tissue differences in cell cycle progression were analyzed by comparing the percentage of cells in S phase between the brain–SG complexes and wing discs. Tissue (brain–SG and wing disc) and larval age (days 10, 13, 16 and 23 after the 5th instar molt) were treated as fixed factors. The results of Tukey's HSD *post hoc* tests are indicated in the figures.

## RESULTS

### Strain differences in diapause parameters

European corn borer larvae either enter diapause at the end of the 5th instar feeding period and prior to pupation in response to short-day and cool temperature conditions, or they develop from larvae to pupae and then reproducing adults under long-day conditions, even in relatively cool temperatures (Fig. 1A). In this study, a long-day treatment of 16 h:8 h L:D at 23±1°C induced 100% non-diapausing individuals in both the BE and UZ strains, while a short-day treatment of 12 h:12 h L:D at 23±1°C induced 55.3% and 100% diapausing larvae in the BE and UZ strains, respectively (Fig. 1C, *P*<0.001). After exposing diapausing individuals to a diapause-terminating, long-day treatment of 16 h:8 h L:D at 26±1°C, 50% of diapausing BE and UZ individuals terminated diapause and pupated at day 12 and day 46, respectively (Fig. 1B).

### Strain differences in cell cycle progression

**Fig. 2.**
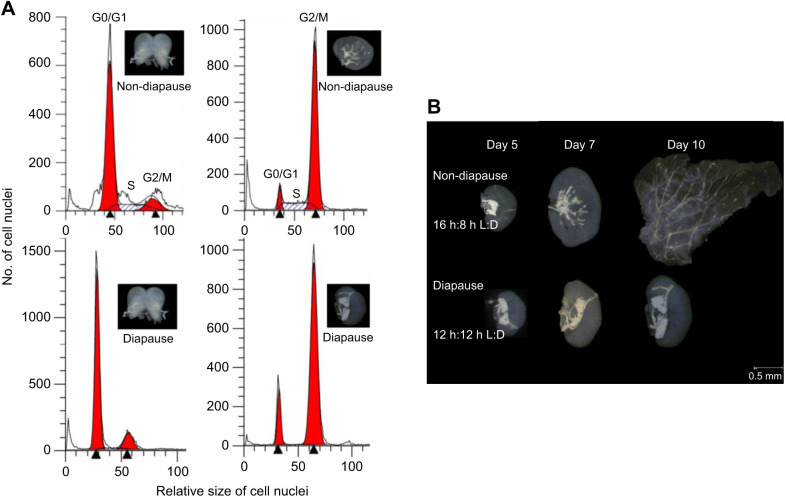
**Cell cycle status of UZ strain *O. nubilalis* larvae brain–subesophageal complexes and wing discs.** (A) Examples of flow cytometry output for brain–subesophageal (SG) complexes and wing discs from diapausing and non-diapausing larvae. Samples were collected on day 7 after the 5th instar molt. The black arrowheads indicate the mean of the relative size of cell nuclei in the G0/G1 and G2/M phases; the areas under the curve representing either G0/G1 or G2 cell cycle phases are highlighted in red. (B) Light microscopy images comparing the development of UZ strain wing discs under long-day (16 h:8 h L:D) and short-day (12 h:12 h L:D) conditions. Days 5, 7 and 10 represent time after molting into the 5th instar.

**Fig. 3.**
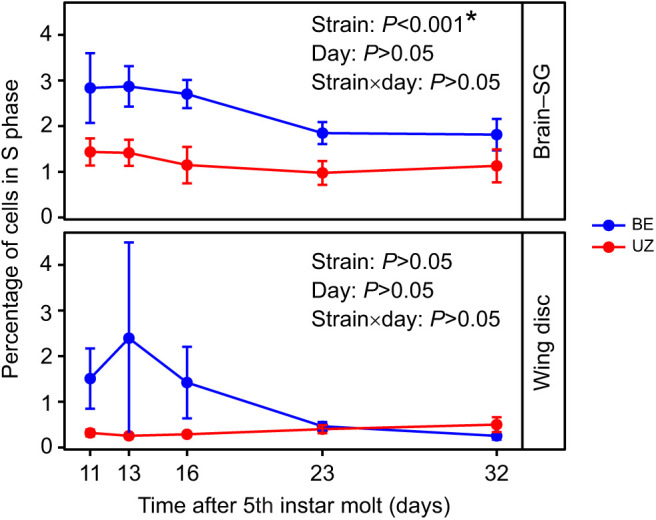
**Comparison of the relative proportion of cells in S phase in the brain–SG complex and wing disc between the two *O. nubilalis* strains during diapause maintenance.** The early-emerging BE strain has significantly more cells in the brain–SG complex in S phase, suggesting faster development, with a similar but non-significant trend in the wing discs (*n*=6 per strain per sampling day, *n*=60 total). Means±s.e.m.; note that some error bars are subsumed within the symbols. *P*-values for each model term are shown within the figure and full models appear in Table 1.

**Fig. 4.**
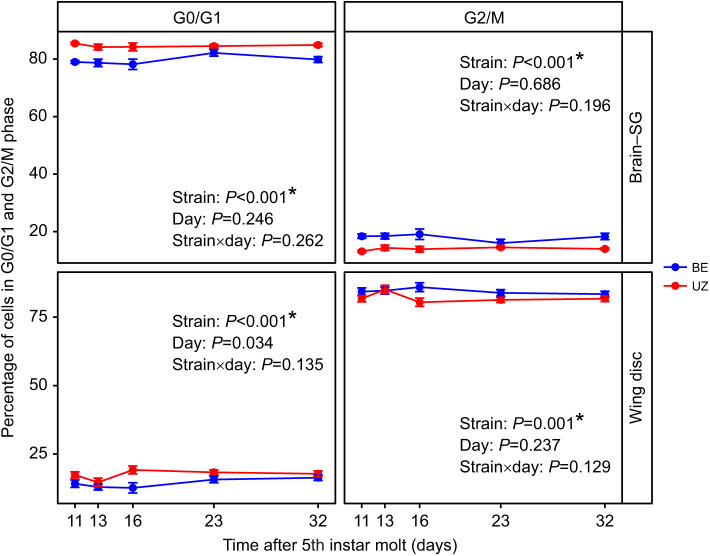
**Comparison of the relative proportion of cells in G0/G1 and G2/M phases in the brain–SG complex and wing disc of the two *O. nubilalis* strains during diapause maintenance.** The proportion of cells in G0/G1 phase is significantly higher in the diapausing UZ strain than in the BE strain in both the brain–SG complex and the wing disc tissues, whereas the proportion of cells in G2/M phase is significantly lower in the diapausing UZ strain than in the BE strain (*n*=6 per strain per sampling day, *n*=60 total). Means±s.e.m.; note that some error bars are subsumed within the symbols. *P*-values for each model term are shown within the figure.

Photoperiod affected cell cycle status in both BE and UZ strains (Fig. S1A; Fig. 2). The number of cells in S phase remained consistently high in tissues of larvae under long-day conditions and decreased to very low levels under short-day conditions. Consistent with other insect systems, a substantial slowdown of the cell cycle is a hallmark of diapause in *O. nubilalis*. Numerous studies have shown that the BE strain terminates larval diapause and resumes pupal development sooner after transfer to long-day conditions than the UZ strain of the European corn borer (Dopman et al., 2005; Glover et al., 1991, 1992; McLeod et al., 1979; Wadsworth et al., 2013). Thus, we expected that the BE strain would suppress its cell cycle less than the UZ strain during the diapause maintenance stage so that the BE strain would resume the cell cycle earlier and resume larval–pupal metamorphosis faster than the UZ strain under diapause-termination conditions. We found no significant differences in the proportion of cells in S phase between the BE and UZ strains for either tissue in non-diapausing larvae (brain–SG: strain, *P*=0.789; wing disc: strain, *P*=0.199). However, in the brain–SG complexes, the proportion of cells in S phase was significantly higher in the BE strain (2.51%) than in the UZ strain (1.43%) during the diapause maintenance phase (Fig. 3, Table 1; strain: *P*<0.001). In wing discs, there was a trend towards the proportion of cells in S phase being higher in the BE strain than in the UZ strain through time during diapause maintenance, but the strain effect did not reach our *a priori* cutoff for significance (Fig. 3, Table 1; strain: *P*>0.05). In addition, the proportion of cells in G0/G1 phase was significantly higher in the diapausing UZ strain than in the BE strain in both the brain–SG complex and the wing disc tissues, whereas the proportion of cells in G2/M phase was significantly lower in the diapausing UZ strain than in the BE strain (Fig. 4, strain: *P*<0.05).

**Table 1.**
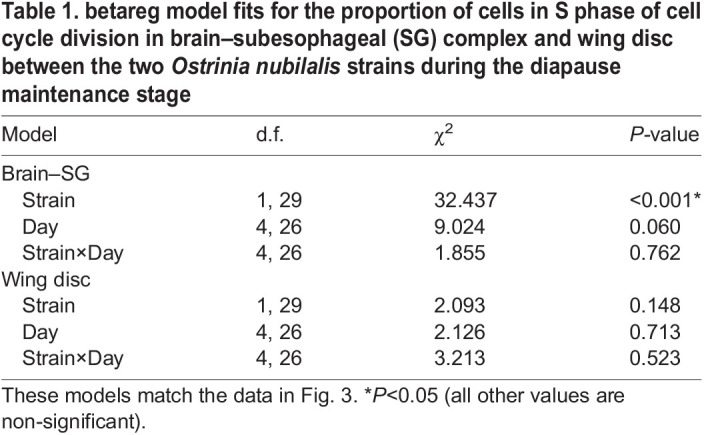
betareg model fits for the proportion of cells in S phase of cell cycle division in brain–subesophageal (SG) complex and wing disc between the two *Ostrinia nubilalis* strains during the diapause maintenance stage

**Fig. 5.**
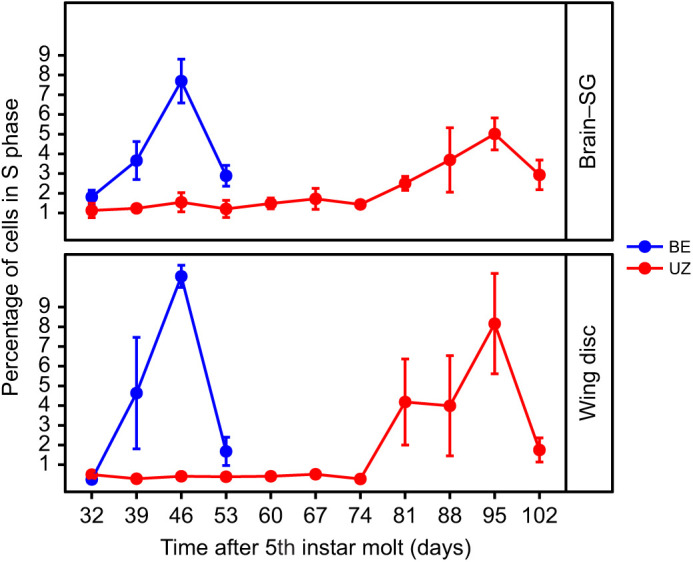
**Comparison of the relative proportion of cells in S phase in the brain–SG complex and wing disc of *O. nubilalis* during diapause termination.** After exposure to long-day conditions to trigger diapause termination, the BE strain restarts cell cycling and development much earlier than the UZ strain (*n*=6 per strain per sampling day, *n*=90 total). Means±s.e.m.; note that some error bars are subsumed within the symbols. *P*-values for each model term and full models appear in Table S2.

After transferring diapausing larvae of both strains from diapause-maintaining conditions (12 h:12 h L:D, 23±1°C) to diapause-termination conditions (16 h:8 h L:D, 26±1°C), the proportion of cells in S phase (DNA synthesis) and G2/M phase (cell division and mitosis) increased earlier in the BE strain than in the UZ strain in both the brain–SG complexes and wing discs (Fig. 5; Fig. S1B). Specifically, the increase in the proportion of cells in S and G2/M phase began within 1 week (day 39) of transfer to diapause-terminating conditions in the BE strain, but not until 7 weeks (day 81) after transfer in the UZ strain (Fig. 5; Fig. S1B).

### Tissue differences in cell cycle progression

**Fig. 6.**
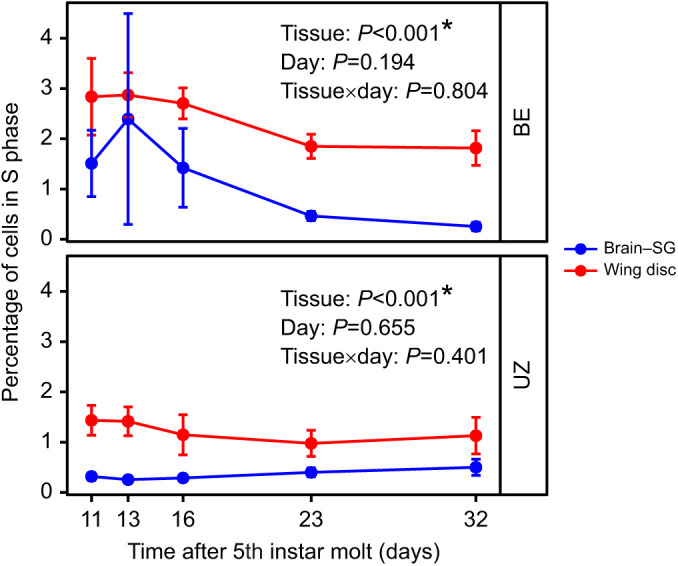
**Comparison of the relative proportion of cells in S phase in the two *O. nubilalis* strains between the brain-SG complex and wing disc.** The brains are engaged in more active development than the wing discs during the diapause maintenance stage (*n*=6 per strain per sampling day, *n*=60 total). Means±s.e.m.; note that some error bars are subsumed within the symbols. *P*-values for each model term are shown within the figure and full models appear in Table 2.

Generally, cells in diapausing larvae of *O. nubilalis* were in G0/G1 or G2/M phase, but the proportion of cells in each phase differed substantially between the brain–SG complexes and wing discs in each strain (Table S2; tissue, G0/G1: *P*<0.001, S: *P*<0.001, G2/M: *P*<0.001). Specifically, in both BE and UZ strains, a large proportion of brain–SG cells (∼80%) were in G0/G1 phase and many fewer cells (∼20%) were in G2/M phase. In contrast, a small proportion of wing disc cells (∼20%) were in G0/G1 phase and most cells (∼80%) were in G2/M phase for both strains (Fig. S1A; Fig. 4). Although the cell cycle slowed down markedly in both tissues of *O. nubilalis* during diapause, the proportion of cells in S phase was significantly higher in the brain–SG complexes than in the wing discs in both strains, leading us to speculate that brain–SG cells may be proliferating slowly, but perhaps at a greater rate than cells in the wing disc (Fig. 6, Table 2; BE strain: *P*<0.001, UZ strain: *P*<0.001).

**Table 2.**
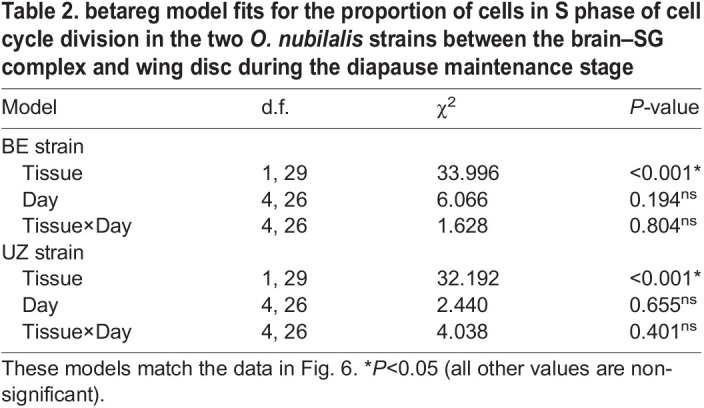
betareg model fits for the proportion of cells in S phase of cell cycle division in the two *O. nubilalis* strains between the brain–SG complex and wing disc during the diapause maintenance stage

The morphological development of the wing discs also clearly reflected how photoperiod affects cell cycle progression. Under long-day conditions, wing discs developed continuously into the general adult wing shape on the day before prepupa formation (day 10 after 5th instar molt) (Fig. 2B). However, under short-day conditions, wing discs remained undeveloped, strengthening the idea that most cells in the wing discs of diapausing larvae of *O. nubilalis* are in a state of cell cycle slowdown.

### Abundance of cell cycle-associated transcripts

**Fig. 7.**
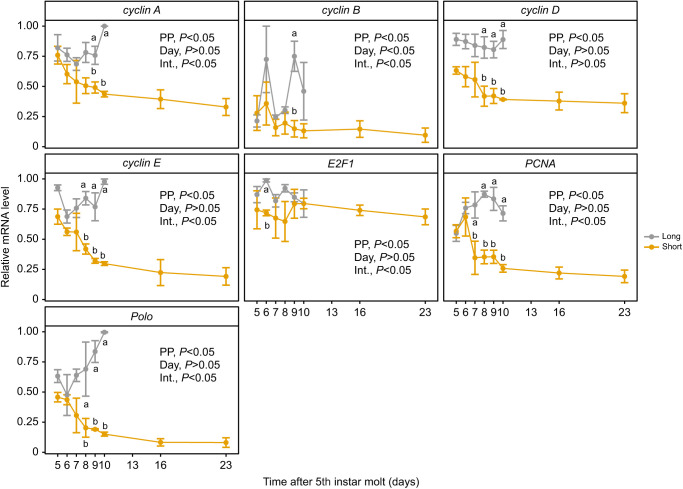
**Ontogenetic profile of transcript abundance of a series of cell cycle regulators in the brain–SG complex of long-day and short-day *O. nubilalis* UZ strain 5th instar larvae.** There is a clear decrease in *cyclin A*, *cyclin B*, *cyclin D*, *cyclin E*, *PCNA* and *polo* transcript abundance upon entry into diapause (*n*=3 per strain per sampling day, *n*=42 total). Results of the beta regression (betareg model) are shown in each panel (PP, photoperiod; Int., interaction). Means±s.e.m.; note that some error bars are subsumed within the symbols. Different letters denote significant differences in transcript abundance between long-day and short-day caterpillars within each time point after Tukey's HSD adjustment for multiple comparisons. *P*-values for models for each transcript and term are shown within the figure and full models appear in Table S3.

We compared transcript abundance for seven cell cycle-associated genes (*PCNA*, *cyclin A*, *cyclin B*, *cyclin D*, *cyclin E*, *E2F1* and *polo*) in long-day, non-diapause and short-day, diapause-destined larvae of the UZ strain of European corn borer as a baseline for comparing the transcript abundance of these same cell cycle genes between the UZ and BE strains during diapause maintenance and termination. The transcript abundance of all the tested cell cycle regulators except for *E2F1* gradually decreased over the diapause-induction trajectory and was significantly lower than that of their non-diapausing counterparts (Fig. 7; Table S3; photoperiod: *P*<0.05).

**Fig. 8.**
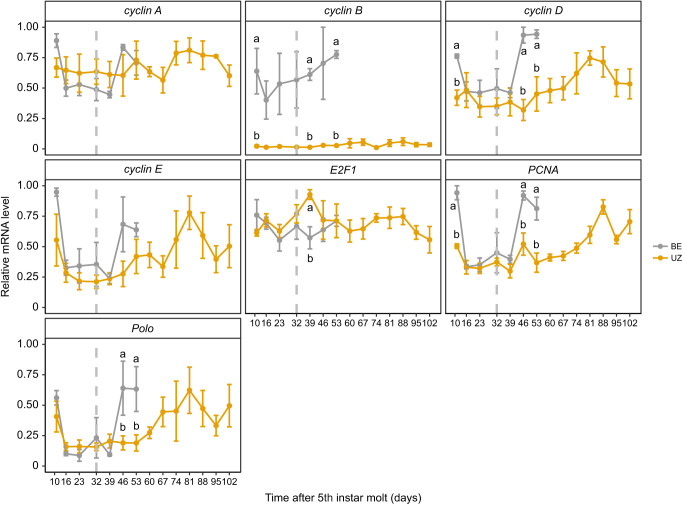
**Ontogenetic profile of transcript abundance of cell cycle regulators in the brain–SG complex of the two *O.  nubilalis* strains during diapause maintenance and termination phases.** The abundance of *cyclin B*, *cyclin D*, *PCNA* and *polo* is higher in the BE strain than in the UZ strain, consistent with our hypothesis of faster brain development in the earlier emerging BE strain (*n*=3 per strain per sampling day, *n*=42 total). Vertical gray dashed lines at day 32 after 5th instar molt indicate the day larvae were transferred from diapause maintenance conditions to diapause termination conditions. Means±s.e.m.; note that some error bars are subsumed within the symbols. Different letters denote significant differences in transcript abundance between long-day and short-day larvae within each time point after Tukey's HSD adjustment for multiple comparisons. *P*-values for models for each transcript are shown in Table S4 for the diapause maintenance phase before the vertical dashed line and in Table S5 for the diapause termination phase after the vertical dashed line.

During the diapause maintenance phase, the transcript abundance of *PCNA* and *cyclin D* was significantly higher in the BE strain than in the UZ strain at our first time point (day 10), then reduced to similar levels to those in the UZ strain for later time points during diapause maintenance. But then *cyclin D* and *PCNA* increased in abundance much more quickly after transfer to diapause-terminating conditions in the BE strain than in the UZ strain, as expected if the BE strain is more developmentally responsive to diapause-terminating cues (Fig. 8; Table S4; strain: *P*<0.05). Interestingly, transcripts for *cyclin B* were more abundant in the BE larvae brain than in the UZ larvae brain throughout the diapause maintenance phase (Fig. 8; Table S5; strain: *P*<0.05). Transcript abundance for all of our focal genes, except for *E2F1*, was upregulated in the BE strain within 1 week of exposure to diapause-terminating, long-day conditions, whereas transcript abundance remained suppressed in the UZ strain for several weeks after transfer (Fig. 8; Table S5). The peaks of cell cycle regulator transcript abundance were at week 2 (day 46) and week 7 (day 81) after exposure to diapause-terminating conditions for the BE and UZ strains, respectively, corresponding to earlier diapause termination in the early-emerging BE strain than the late-emerging UZ strain.

## DISCUSSION

Shifts in diapause timing have rapidly evolved across latitudinal gradients in the European corn borer after range expansion from Europe to North America in ∼1910 (Caffrey and Worthley, 1927; Glover et al., 1992; McLeod and Beck, 1963; Rabb and Kennedy, 1979). The earlier emerging BE strain and later emerging UZ strain differ by ∼30 days in the length of the diapause termination phase (Wadsworth et al., 2013). Understanding the proximate causes of shifts in seasonal timing via diapause facilitates prediction of future responses and persistence in the face of anthropogenic change (Razgour et al., 2019). Because cell cycle slowdown is a hallmark of diapause, in this paper we explored associations between cell cycle progression and regulation with divergence in diapause termination timing in the European corn borer.

### Strain comparisons in cell cycle progression

Insect diapause is characterized by a major slowdown of the cell cycle, and the resumption of substantial cell proliferation is indicative of diapause termination (Denlinger, 2002; Hand et al., 2016; Koštál et al., 2009; Ragland et al., 2011; Shimizu et al., 2016; Tammariello and Denlinger, 1998). Instead of completely arresting the cell cycle, both the brain–SG complex (e.g. in the UZ strain, S phase: 1.43%) and wing discs (S phase: 0.38%) of diapausing larvae still showed some nuclei that were consistent with low levels of cell division. The proportion of brain–SG cells in S phase was significantly lower in the later emerging UZ strain than in the earlier emerging BE strain during the diapause maintenance stage, suggesting that the faster post-winter diapause termination observed in the BE strain may be facilitated by higher baseline rates of slow cell division during the larval diapause maintenance period. We hypothesize that with a more actively proliferating neural system, the BE strain might either be more sensitive to diapause-termination cues than the UZ strain or respond more quickly to diapause-termination cues, or a combination of the two. Furthermore, a relatively higher level of cell division in the brain–SG complex in the earlier emerging BE strain might enable faster diapause development and earlier post-winter diapause termination compared with the later emerging UZ strain. Measuring cell proliferation rates in both BE and UZ strains during the diapause maintenance phase and after winter during the diapause-termination phase will be needed to test this hypothesis; for example, with an EdU (5-ethynyl-2′-deoxyuridine) incorporation assay. Corresponding to the higher baseline proportion of nuclei in S phase during the larval diapause period in the BE strain, transcript abundance of cell cycle regulators (*cyclin B*, *cyclin D* and *PCNA*) was significantly higher in the BE strain than in the UZ strain during the diapause maintenance phase (Fig. 8). Taken together, these data support the idea that although cell proliferation rates are significantly slowed down during diapause in both strains, the earlier emerging BE strain has slightly faster rates of development during the diapause-maintenance phase.

The divergence in life cycle timing between the earlier emerging BE strain and later emerging UZ strain of *O. nubilalis* has previously been attributed to a delay in the timing of larval diapause termination wherein the earlier emerging BE strain has a shorter period of post-winter developmental suppression (Wadsworth et al., 2013). Consistent with this previous study, our results also showed earlier cell cycle resumption in the BE strain compared with the UZ strain. Specifically, the proportion of cells in S phase started to increase rapidly in the earlier emerging BE strain at week 1 (day 39) and peaked at week 2 (day 46) after transfer to diapause-termination conditions, whereas cell proliferation in the later emerging UZ strain remained suppressed until week 7 (day 81) and peaked at week 9 (day 95) after transfer to diapause-terminating conditions (Fig. 5). The proportion of cells in S phase decreased at week 3 (day 53) for the BE strain and at week 10 (day 102) for the UZ strain, suggesting the existence of some deeply diapausing individuals in both strains that had not terminated diapause along with the majority of the population. This difference in the timing of cell cycle resumption corresponds to the average diapause termination timing between BE and UZ strains, demonstrating that an earlier resumption of cellular proliferation in the BE strain than the UZ strain is associated with the divergence in life cycle timing between the two strains of *O. nubilalis*.

Earlier transcriptomic studies of diapause development between these two strains of the European corn borer have shown a rapid increase in the abundance of cell cycle-associated transcripts upon exposure to diapause termination conditions in the BE strain but not in the UZ strain (Wadsworth and Dopman, 2015). In agreement with our data, Wadsworth and Dopman (2015) showed that genes involved in active cell cycling (*cyclin A*, *cyclin B*, *Cdk4*, *polo*, and *PCNA*) were upregulated within days (day 1 and day 7) of long-day exposure in the BE strain, but remained at low abundance in the UZ strain on day 1 and day 7 after long-day exposure. Generally, the complexes of cyclin D and its partners Cdk4 and Cdk6 phosphorylate the pocket pRB proteins, and then the phosphorylated pRB proteins dissociate from transcription regulator E2F1 (Attwooll et al., 2004). Afterwards, the expression of cyclin A and cyclin E, which are critical for the S phase and G1–S transition, respectively, is activated (Attwooll et al., 2004). Additional studies show that cyclin D and cyclin E are required to initiate entry into the cell cycle (Jackson et al., 1995; Edgar and Lehner, 1996).

In this study, we measured the expression of cell cycle regulators (*cyclin A*, *cyclin B*, *cyclin D*, *cyclin E*, *E2F1*, *polo* and *PCNA*) in the brain by sampling weekly after exposure to diapause termination conditions in both strains (Fig. 8). As expected, the transcript levels of *cyclin D* and *PCNA* were upregulated after 1 week of exposure to diapause-terminating conditions in brain–SG tissue of BE strain diapausing caterpillars, but not the UZ strain, a pattern consistent with earlier diapause termination in the BE strain than the UZ stain.

The expression profiles for most of our focal genes differed only in the timing of the increase in transcript abundance after the onset of long-day cues. However, the pattern of *cyclin B* transcript abundance stood out as being higher in the earlier emerging BE strain than in the later emerging UZ strain throughout the diapause maintenance period. Cyclin B is one of the major gatekeepers of the transition from G2 phase to mitosis and the complex formation of cyclin B with Cdk1 is under the control of upstream factors including Polo kinase and other kinases, such as Myt1 and Wee1 (Sanchez et al., 2003). The greater abundance of *cyclin B* transcripts throughout the diapause maintenance phase in the BE strain than in the UZ strain further reinforces our speculation that either (a) the BE strain is primed for faster resumption of the cell cycle than the UZ strain after diapause-terminating cues are sensed, or (b) the brains of BE strain larvae are undergoing slightly higher rates of slow movement through the cell cycle than the brains of UZ larvae during this same period. Either alternative could lead to faster completion of diapause and earlier resumption of development in the BE strain than the UZ strain.

The upstream events that might regulate an earlier or later resumption of the cell cycle in response to diapause-terminating temperature and day length cues, and therefore a difference in the timing of diapause termination, are still unclear and must be investigated. Mitogen expression can activate a series of cellular regulation genes leading to entry into S phase (Adhikary and Eilers, 2005). For example, in *Drosophila melanogaster*, Wingless, a member of the Wnt signaling pathway, may act as a mitogen that governs the proliferation of imaginal disc cells and patterning of future adult structures (Edgar and Lehner, 1996; Swarup and Verheyen, 2012). In the apple maggot *Rhagoletis pomonella*, the Wnt signaling pathway has also been nominated as a potential upstream candidate for regulation of diapause termination (Dowle et al., 2020; Ragland et al., 2011). Similarly, in European corn borers, many transcripts involved in the Wnt signaling pathway increased in abundance in the BE strain but remained at low abundance in the UZ strain upon exposure to diapause-terminating, long-day conditions (Wadsworth and Dopman, 2015). Here, we propose that the Wnt signaling pathway is involved in regulating the timing of diapause termination by promoting proper patterns of growth and development in insects (metamorphosis) via cell cycle regulation and cellular communication (Logan and Nusse, 2004; Gokhale and Shingleton, 2015; Wadsworth and Dopman, 2015). Of course, this hypothesis will require substantial future work to test. Furthermore, how photoperiodic information is transduced into changes in mitogen expression and further diapause timing remains a mystery. Circadian clock genes have been abundantly studied to fill the gap between photoperiodic cues and diapause regulation. Findings from Kozak et al. (2019) provide insightful evidence of circadian clock gene regulation of diapause seasonal timing, specifically that allelic variation in two circadian clock genes, *period* (*per*) and *pigment-dispersing factor receptor* (*Pdfr*), are causal to the evolution of diapause timing in the European corn borer (Kozak et al., 2019). Larval diapause is regulated by a suppression of ecdysteroid production and release from the prothoracic gland with stimulation of prothoracicotropic hormone PTTH, whose production is regulated by the circadian clock via the indolamine metabolism pathway in non-diapause development (Denlinger, 2002; Denlinger et al., 2012; Wang et al., 2013). Also, cell division of insect tissues, such as imaginal discs, is ecdysone dependent (Koyama et al., 2004). Therefore, allelic variation in *per* and *Pdfr* may shift the timing of diapause termination by influencing levels of hormone production and secretion as well as cellular progression, although this hypothesis remains to be tested. To fully understand what molecular mechanisms of diapause regulation have facilitated the evolution of temporal divergence by diapause timing in the early- and late-emerging strains of European corn borer, the mechanistic hypotheses about cell cycle regulation above must ultimately be investigated in future studies done in the laboratory and extend to ecologically relevant conditions in nature.

### Tissue comparisons of cell cycle progression

Our results are consistent with previously published reports of diapausing individuals having the majority of their cells in G0/G1 or G2 phase (Nakagaki et al., 1991; Champlin and Truman, 1998; Tammariello and Denlinger, 1998; Koštál et al., 2009; Shimizu et al., 2016). However, one of the ways our work stands out as novel is that we have studied two different tissues within the same individual, the brain–SG complex and the wing disc. By directly comparing tissues within the same individuals, we have shown that the stage of cell cycle slowdown is not consistent among tissues within a single diapausing individual. Specifically, we found that cells of the brain–SG complex were predominantly in the G0/G1 stage of the cell cycle, as has been reported for the brains of diapausing pupae in the flesh fly *S. crassipalpis* (Tammariello and Denlinger, 1998), and the brain–SG complexes of diapausing larvae of the drosophilid fly *C. costata* (Koštál et al., 2009). In contrast, wing disc cells occurred predominantly in the G2 stage of the cell cycle, consistent with the optic lobes of diapausing pupae in the tobacco hornworm, *M. sexta* (Champlin and Truman, 1998).

The larval wing imaginal disc is an epithelial-derived sheet of undifferentiated cells that develops into the adult wing during metamorphosis (Bryant, 1975; Beira and Paro, 2016). Non-diapausing 5th instar larval wing discs undergo dramatic changes in size and shape (Fig. 2), supported by active cell division, with most cells in the G2/M phase (∼80%) ready for mitosis. In contrast to wing discs, the brain–SG complex is mostly developed during late 5th instar larval stage; thus, cell division was less active with fewer cells in G2/M phase in the brain–SG complex (∼16%). Although cells of the brain–SG complex and wing discs undergo canonical cell cycles (G1→S→G2→M), tissues with different characteristics and functions may be under the control of tissue-specific cell cycle regulatory proteins (Boonstra, 2003). One proximate mechanistic possibility for the distinct differences in the cell cycle phase we observed between the brain–SG complex versus wing discs may be differences in expression of G1 or G2 phase cyclin/Cdk in each tissue. Molecular mechanisms underlying cell cycle arrest have been investigated in only a few diapausing insect species. Most of the studies to date have focused on the cell cycle regulation protein complex (cyclins and Cdks) and proteins that are known to regulate cyclins and cdks, such as p53, p21, cdc25, etc. (Schafer, 1998). Generally, the expression of proliferating cell nuclear antigen (PCNA), a δ DNA polymerase cofactor, is consistently down-regulated in the tissues of diapausing individuals (Bao and Xu, 2011; Huang et al., 2015; Koštál et al., 2009; Ragland et al., 2011; Shimizu et al., 2016; Tammariello and Denlinger, 1998). However, the expression patterns of transcripts for other regulatory proteins, such as cyclin D, cyclin E, p21, p53, Wee1 and Myt1 kinases, cdc25 phosphatase (String) and Dacapo (p27), differ across species during diapause. Taking cyclin D and cyclin E as examples, the relative levels of both genes were barely influenced by photoperiodic regime for inducing diapause or direct development in brains of *C. costata* (Koštál et al., 2009), but decreased during diapause in brains of *N. vitripennis* (Shimizu et al., 2016). The proximate mechanisms underlying the differences in the cell cycle phases in the brain–SG complex versus wing discs during diapause remain unknown. Another difference between the brain–SG and wing disc tissues may be the number of cells that are fully differentiated. The flow cytometry methods we used cannot distinguish between cells that will proliferate in the future but are currently in G0/G1 versus fully differentiated cells that will no longer undergo division. We expect that the larval brain has a fair number of both cells that will proliferate at larval–pupal metamorphosis and cells that are in a fully differentiated state, including the population of brain–SG cells that will undergo programmed cell death at larval–pupal metamorphosis (Fahrbach, 1997; Levine and Truman, 1982), whereas wing discs almost completely consist of imaginal stem cells that will undergo rapid proliferation and differentiation upon larval–pupal metamorphosis (Nijhout et al., 2007). A detailed study of the abundance and manipulation of cell cycle regulatory proteins is clearly needed in both brain–SG and wing discs of *O. nubilalis* in the future to settle this question.

In addition to our desire to develop an understanding of the proximate mechanisms underlying the massive developmental slowdown that is characteristic of insect diapause, an outstanding question is whether there is any ultimate, or selective, benefit to having cells slow down in the G0/G1 or G2 stages of the cell cycle during diapause. Cells that are arrested in the G2 stage can ultimately undergo mitotic division more rapidly than cells that are in the G0/G1 stage. We note that the relative change in size of the wing disc at larval–pupal metamorphosis is much greater than the relative change in size of the brain in European corn borers and many other holometabolous insects (Nijhout, 2011; Nijhout et al., 2013). Given our observation that cells in the wing discs of diapausing European corn borer larvae were largely in the G2 stage whereas cells in the brain were largely in the G0/G1 stage, we speculate that tissues requiring faster proliferation and relative growth may endure their diapause slowdown in a later stage of the cell cycle. This explanation may be further extended to potentially understand differences in the stage of cell cycle slowdown across species. Future work will be needed to build evidence for or against our speculation that tissues or species that have their cells in G2/M can resume proliferative development faster than those in G0/G1 during diapause. Furthermore, it is very much possible that the differences we observe across tissues or across species in the stage of the cell cycle during diapause are largely driven by other aspects of developmental processes that are not directly the result of selection on the rate of resuming development or may not be due to selection at all and rather may be a product of unknown constraints on cell cycle regulation across tissues or taxa.

Additionally, our observation of only low levels of S phase cells in both brain–SG complexes and wing discs reinforces the idea that diapause is actually not a state of complete developmental arrest, as has been put forth by several previous authors as detailed below. Instead, we think it useful to envisage diapause as a programmed slowdown of development. For example, in the pea aphid, *Acythosiphon pisum*, diapausing embryos showed evident cell division and leg growth during the diapause maintenance phase, but at a much reduced rate compared with non-diapause embryos (Shingleton et al., 2003). Also, active mitotic activity was discovered in diapausing eggs of *D. nigrofasciatus* with approximately 5% of their cells in S phase (Shimizu et al., 2018). Continued morphological development during diapause has also been reported in *Austroicetes cruciata* (Andrewartha, 1943), *Cirphus unipunctata* (Saulich, 1975) and *Sesamia nonagriodes* (Gadenne et al., 1997). Thus, the field of diapause regulatory biology may benefit from a shift in thought about whether diapause is really a state of developmental arrest or just a massive, regulated slowdown of development in target tissues. That said, we do acknowledge that our current data on the phases of the cell cycle only provide static snapshots of the proportion of cells that are in each cell cycle phase and additional work will be needed using techniques that can more directly estimate rates of cell cycling over the course of diapause.

The brain–SG complexes and wing discs also suppressed cell division to different levels during diapause. The proportion of cells in S phase was significantly higher in brain–SG complexes during diapause than in the wing discs of diapausing larvae. From an energetic perspective, diapausing animals generally reduce unnecessary costs to save energy reserves (Hahn and Denlinger, 2011; Sinclair, 2015). However, diapausing animals also selectively maintain the activity of some tissues, such as the brain, to survive diapause and coordinate their development with diapause-termination cues (Hahn and Denlinger, 2011; Sinclair, 2015). Insect brains are the sensory neural center that receives diverse environmental stimuli and makes responses by controlling physiology and behaviors (Wehner, 2003; Srinivasan, 2010; Warrant and Dacke, 2010; Chittka and Skorupski, 2011; Menzel, 2012). Because neural tissue is still metabolically costly to maintain, brains of diapausing insects can suppress the cell cycle partially by selectively shutting down the development of sensory structures such as neuropils related to olfactory rather than light sensing, which might be critical for sensing photoperiod (Lehmann et al., 2017). However, flight ability is not necessary until the adult stage; thus, cellular proliferation in wing discs of diapausing larvae of *O. nubilalis* can be largely suppressed during diapause to save energy.

### Conclusions

Our study suggests that diapause in larvae of the European corn borer is characterized by a state of cell cycle slowdown in target tissues such as brains and wing discs. Divergence in cell cycle progression is associated with shifts in life cycle timing via diapause regulation between the early-emerging BE and late-emerging UZ strains. Further work on the upstream regulation of cell cycle progression is clearly needed to understand the molecular basis of diversification by temporal isolation and, further, to predict adaptation of phenology in response to continued global climate change.

## Supplementary Material

10.1242/jexbio.245244_sup1 Supplementary information
