# Supplementary material for: Divergence in cell cycle progression is associated with shifted phenology in a multivoltine moth: the European corn borer, Ostrinia nubilalis
Source: J Exp Biol. 2023 Jun 9;226(11):jeb245244. doi: 10.1242/jeb.245244 (PMC10281267; doi:10.1242/jeb.245244)
Supplement: Supplementary information [file jexbio-226-245244-s1.pdf]

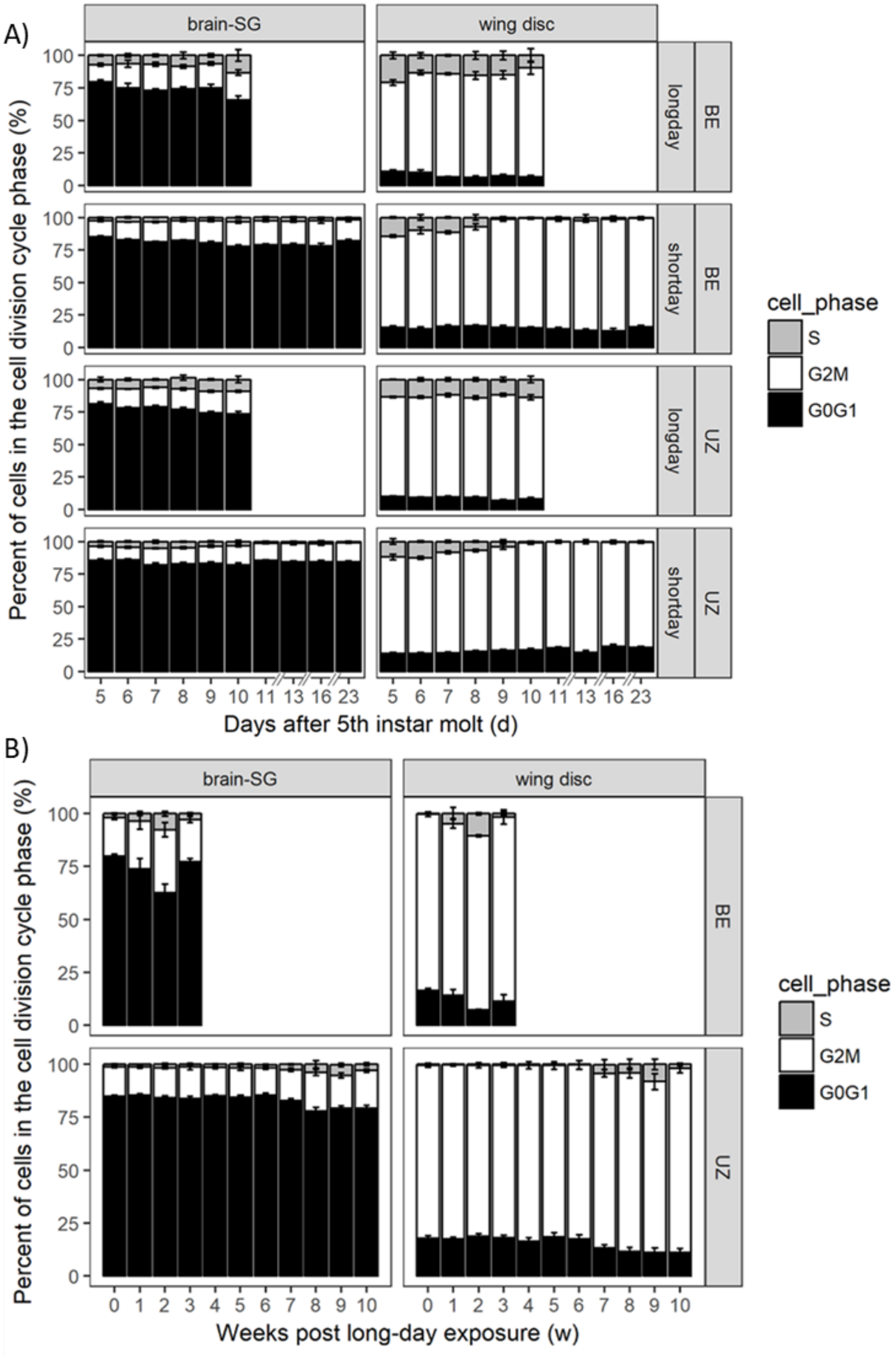

Fig. S1. Overall descriptions of the proportion of cells in each tissue in each phase of the cell cycle A) from long-day and short-day conditions and B) after long-day exposure between the two strains. The top of each box represents the mean and bars represent the standard error, although these bars are sometimes subsumed within the symbols.

Journal of Experimental Biology • Supplementary information

Table S1. Oligonucleotides used for qRT-PCR.

Gene coding for	Forward (5′–3′)	Reverse (5′–3′)
PCNA	GGACAACTCTCACGTATCTCTTG	CGGTGCTAGGTAGTAACGAATG
cyclin A	CACTCTGCTGTCCATACTCATC	GCATCCTATCGATGTCCATCTC
cyclin B	GGGAATCTTCGCCAGTTCATAG	CATGTTCATCGCCAGCAAATAC
cyclin D	CTCATCTTCTACACAGCCAACA	AGTACAGCGTGAACTGGTAATC
cyclin E	TGTTTGACAGGCACCCTAATC	GCAGCTATGAAGAGACACGTTAT
E2F1	AACGGACGACATCGACAATC	GAGCCTTTCCACTTCACTATCC
polo	TGGCCCTTCTTCGTCAATATC	TTGGCTTCCACAGCTTCTT
RPS03	ATACAAACTTATCGGAGGTCTCG	ATGATCTTTACCTTGATTCCAAGC

Table S2. Betareg model fits for proportions of cells in G0/G1, G2/M, and S phases of cell cycle division after exposure to diapause-termination conditions between the two strains through time. Data of proportions of cells for each cell cycle phase from days 32, 39, 46 and 53 after 5th instar molt were analyzed. These models match with the data in Fig. 5.

Model	Df	Chisq	P_value
Brain-SG × G0/G1 phase			
Strain	1, 23	55.929	<0.000*
Day	3, 21	23.692	<0.000*
Strain*Day	3, 21	13.854	0.003*
Brain-SG × G2/M phase			
Strain	1, 23	48.121	<0.000*
Day	3, 21	14.021	0.003*
Strain*Day	3, 21	9.290	0.026*
Brain-SG × S phase			
Strain	1, 23	43.956	<0.000*
Day	3, 21	31.073	<0.000*
Strain*Day	3, 21	10.109	0.018*
Wing disc × G0/G1 phase			
Strain	1, 23	30.475	<0.000*
Day	3, 21	7.371	0.0614^{ns}
Strain*Day	3, 21	14.683	0.002*
Wing disc × G2/M phase			
Strain	1, 23	4.722	0.030*
Day	3, 21	5.853	0.119^{ns}
Strain*Day	3, 21	7.654	0.054^{ns}
Wing disc × S phase			
Strain	1, 23	18.647	<0.000*
Day	3, 21	45.382	<0.000*
Strain*Day	3, 21	15.931	0.001*

ns, non-significant. Starred terms are significant at *P<0.05*.

Table S3. Betareg model fits for transcript abundance of a series of cell cycle regulators (*cyclin A*, *cyclin B*, *cyclin D*, *cyclin E*, *E2F1*, *PCNA* and *polo*) in the brain-SGs of long-day and short-day 5th instar larvae of UZ strain of *Ostrinia nubilalis*. Data of transcript abundance for each gene from days 5, 6, 7, 8, 9, and 10 were analyzed. These models match with the data in Fig. 7.

Model	Df	Chisq	P_value
cyclin A			
Photoperiod	1,17	32.811	<0.000*
Day	5,13	10.684	0.058 ns
Photoperiod *Day	5,13	38.463	<0.000*
cyclin B			
Photoperiod	1,17	17.179	<0.000*
Day	5,13	16.308	0.006*
Photoperiod *Day	5,13	21.640	<0.000*
cyclin D			
Photoperiod	1,17	55.322	<0.000*
Day	5,13	5.701	0.336 ns
Photoperiod *Day	5,13	10.992	0.052 ns
cyclin E			
Photoperiod	1,17	51.911	<0.000*
Day	5,13	7.030	0.218 ns
Photoperiod *Day	5,13	41.161	<0.000*
E2F1			
Photoperiod	1,17	7.134	0.007*
Day	5,13	7.019	0.219 ns
Photoperiod *Day	5,13	16.790	0.005*
PCNA			
Photoperiod	1,17	17.955	<0.000*
Day	5,13	9.990	0.076 ns
Photoperiod *Day	5,13	34.500	<0.000*
polo			
Photoperiod	1,17	41.178	<0.000*
Day	5,13	7.278	0.201 ns
Photoperiod *Day	5,13	42.459	<0.000*

ns, non-significant. Starred terms are significant at $P<0.05$.

Journal of Experimental Biology • Supplementary information

Table S4. Betareg model fits for transcript abundance of a series of cell cycle regulators (*cyclin A*, *cyclin B*, *cyclin D*, *cyclin E*, *E2F1*, *PCNA* and *polo*) in the brain-SGs of UZ and BE strain larvae of *Ostrinia nubilalis* during the diapause maintenance phase. These models match with the data to the left of the vertical dashed line in Fig. 8 (days 10, 16, 23 and 32 after 5th instar molt), representing the diapause-maintenance phase.

Model	Df	Chisq	P_value
cyclin A			
Strain	1, 11	0.012	0.912^{ns}
Day	3, 9	9.317	0.025^{ns}
Strain *Day	3, 9	16.989	<0.000*
cyclin B			
Strain	1, 11	22.654	<0.000*
Day	3, 9	3.212	0.360^{ns}
Strain *Day	3, 9	2.835	0.418^{ns}
cyclin D			
Strain	1, 11	5.905	0.015*
Day	3, 9	5.164	0.160^{ns}
Strain *Day	3, 9	4.214	0.239^{ns}
cyclin E			
Strain	1, 11	6.706	0.010*
Day	3, 9	27.632	<0.000*
Strain *Day	3, 9	9.238	0.026*
E2F1			
Strain	1, 11	0.778	0.378^{ns}
Day	3, 9	4.999	0.172^{ns}
Strain *Day	3, 9	15.018	0.002*
PCNA			
Strain	1, 11	7.859	0.005*
Day	3, 9	28.380	<0.000*
Strain *Day	3, 9	38.903	<0.000*
polo			
Strain	1, 11	0.001	0.981^{ns}
Day	3, 9	32.510	<0.000*
Strain *Day	3, 9	3.653	0.302^{ns}

ns, non-significant. Starred terms are significant at $P<0.05$.

Table S5. Betareg model fits for transcript abundance of a series of cell cycle regulators (*cyclin A*, *cyclin B*, *cyclin D*, *cyclin E*, *E2F*, *PCNA* and *polo*) in the brain-SGs of UZ and BE strain larvae of *Ostrinia nubilalis* during the diapause termination phase. These models match with the data to the right of the vertical dashed line in Fig. 8 (days 32, 39, 46 and 53 after 5th instar molt), representing the diapause-termination phase.

Model	Df	Chisq	P_value
cyclin A			
Strain	1, 11	0.425	0.515 ^{ns}
Day	3, 9	7.622	0.054 ^{ns}
Strain *Day	3, 9	4.453	0.216 ^{ns}
cyclin B			
Strain	1, 11	34.167	<0.000*
Day	3, 9	7.093	0.069 ^{ns}
Strain *Day	3, 9	5.381	0.146^{ns}
cyclin D			
Strain	1, 11	25.570	<0.000*
Day	3, 9	16.979	<0.000*
Strain *Day	3, 9	30.244	<0.000*
cyclin E			
Strain	1, 11	6.490	0.011*
Day	3, 9	9.409	0.024*
Strain *Day	3, 9	4.758	0.190^{ns}
E2F1			
Strain	1, 11	11.801	0.001*
Day	3, 9	2.210	0.530^{ns}
Strain *Day	3, 9	7.184	0.066^{ns}
PCNA			
Strain	1, 11	17.958	<0.000*
Day	3, 9	20.395	<0.000*
Strain *Day	3, 9	19.427	<0.000*
polo			
Strain	1, 11	8.089	0.004*
Day	3, 9	10.414	0.015*
Strain *Day	3, 9	14.398	0.002*

ns, non-significant. Starred terms are significant at $P<0.05$.

Journal of Experimental Biology • Supplementary information
